# Biological functions of extracellular vesicle double C2-like domain beta in cervical cancer

**DOI:** 10.1038/s41598-024-84643-2

**Published:** 2025-01-02

**Authors:** Sangavi Eswaran, Samatha Bhat, Dinesh Upadhya, Roshan Mascarenhas, Shama Prasada Kabekkodu

**Affiliations:** 1 Department of Cell and Molecular Biology, Manipal School of Life Sciences, Manipal Academy of Higher Education, Manipal, Karnataka 576104 India; 2 Centre for Molecular Neurosciences, Kasturba Medical College, Manipal Academy of Higher Education, Manipal, Karnataka 576104 India; 3 Newcastle University Medicine Malaysia (NUMed), 79200 Johor Bahru, Malaysia; 4 Manipal Center for Biotherapeutics Research, Manipal Academy of Higher Education, Manipal, Karnataka 576104 India

**Keywords:** Cervical cancer, DOC2B, Extracellular vesicles, Calcium, EMT, Senescence, Cancer, Cell biology, Molecular biology

## Abstract

Double C-2 Like Domain Beta (DOC2B) located at 17q13.3 prevents metastasis by senescence induction and epithelial to mesenchymal transition inhibition in cervical cancer (CC). The extracellular vesicle (EV) mediated trafficking of DOC2B and its impact on tumor suppressive activity are not investigated in CC. Using a retroviral method, we first ectopically expressed DOC2B in SiHa, which do not normally express DOC2B. DOC2B-SiHa and vector-SiHa EVs were co-incubated separately with recipient cell and subjected to various cellular and biochemical experiments. For the first time, we demonstrated that DOC2B localizes to EVs, and its transfer to EV may require intracellular calcium. Co-culture of SiHa and HeLa cells with DOC2B-SiHa derived EVs induced morphological changes and suppressed their growth and migration, possibly by induction of G0/G1 to S phase arrest and anoikis. DOC2B-SiHa EVs elevated intracellular reactive oxygen species (ROS) and calcium levels and promoted lipid droplet accumulation and lipid peroxidation rate in recipient cells. DOC2B-SiHa EVs reduced active AKT1 and ERK1/2 levels and EMT marker expression and enhanced cellular senescence and cytotoxic effects of cisplatin. Re-expression of DOC2B significantly altered the global metabolite profile of EVs.  Finally, we demonstrated that intracellular calcium chelation significantly reduces DOC2B localization to EVs and impacts its tumor-suppressive properties. Altogether, EV-mediated DOC2B transfer may reduce the aggressive behavior of CC cells.

## Introduction

Cancer of the cervix (CC) ranked 4^th^ most frequent gynecological cancer worldwide, with 604,127 new cases and 342,000 deaths in 2020. As per GLOBOCAN 2020, CC is the 3^rd^ most frequently diagnosed cancer (123,907 cases; incidence rate: 18.3%) and 2^nd^ leading cause of mortality (mortality rate: 9.1%) in India^1,2^. Sadly 6–29% of all female cancer cases were attributed to CC and showed an increasing trend in the younger population in India^3^. Introducing the HPV vaccine and Pap testing helped reduce global CC cases^4,5^. However, the incidence of CC is still very high in several developing and underdeveloped nations, with a 5-year overall survival of 40–50%^6^. Lack of understanding of the molecular events, advanced-stage identification, and limited therapy choices for late-stage cancer may contribute to a high mortality rate of CC^7^. Therefore, to better manage CC, it is imperative to fully understand the cellular and molecular processes that occur during carcinogenesis.

Membrane-bound particles called extracellular vesicles (EVs) are expelled into the extracellular space by cells under both normal and pathological situations^8^. Exosomes are EVs that play vital roles in intracellular communication through their cargoes^9^. Previous studies have demonstrated a robust tumor-promoting function for CC-derived exosomes^10,11^. Tumor-derived exosome cargos showed a positive correlation with CC progression^12–14^. miR-146a-5p, miR-151a-3p, miR-125a-5p, and miR-2110 showed significantly higher expression in plasma-derived exosomes isolated from CC patients than in control subjects^15,16^.

CC-derived exosomes enhanced proliferation, growth, migration, invasion, angiogenesis, metastasis, and therapy resistance^12^. Exosomal miR-221-3p, miR-663b, and Wnt2B showed tumor-promoting functions in CC^13,14^. Exosomes derived miR-221-3p from CC promoted endothelial cell proliferation, migration, and angiogenesis by targeting mitogen-activated protein kinase-10 and thrombospondin-2^13^. Exosome miR-663b promoted epithelial-to-mesenchymal transition (EMT) by targeting mannoside acetyl-glucosaminyltransferase-3 to enhance metastasis in CC^14^. Another finding demonstrated that HPV oncoproteins such as E6 and E7 can alter the exosomal cargo, and the extent of exosomal release may promote CC^17^. Thus, tumor-derived exosomes play an active role in the progression of CC pathogenesis. Thus, profiling the exosomal cargo can be a sensitive and specific diagnostic and prognostic indicator for CC.

Double C2-like domain-containing protein Beta (DOC2B), located at Chr.17p13.3, is a member of the double C2-like beta family and is documented for its role in intracellular vesicle trafficking, exocytosis, and neurotransmitter release^18,19^. CC, migratory breast cancer cells, and diabetes are a few diseases that have reported differential expression of DOC2B^18,20,21^. Gastric cancer patients harbored the GARNL4/DOC2B fusion^22^. Previous studies from our group established DOC2B as a tumor growth suppressor protein in CC. We showed that DOC2B downregulation in CC correlated with promoter hypermethylation^23^. The overexpression and knockdown studies have confirmed that DOC2B (i) is a negative regulator of the growth, proliferation, invasion and migration of CC cells, (ii) suppresses metastasis and invasion by inhibiting EMT and inducing senescence, (iii) inhibits the Wnt/β-catenin signaling pathway, and (iv) increases intracellular calcium to retard EMT and senescence induction^18,24^. More recently, we showed that DOC2B increases intracellular calcium levels, oxidative stress, and lipotoxicity with simultaneous structural and functional changes in mitochondria^25^. However, the role of DOC2B present in EVs was elusive.

Here, we examined the tumor suppressive functions of extracellular vesicle DOC2B and explored the potential mechanism in CC. We found that EVs contain DOC2B. EV-mediated DOC2B transfer to SiHa cells (i) increased EV numbers, (ii) reduced growth and migration, and induced apoptosis and G0/G1 to S phase arrest in recipient cells, (iii) enhanced reactive oxygen species (ROS) levels, lipid droplet accumulation, and lipid peroxidation rate, (iv) reduced active forms of AKT1 and ERK1/2, and expression of EMT markers, and (v) enhanced cellular senescence and cytotoxic effects of cisplatin. Finally, we demonstrated that intracellular calcium chelation significantly reduces DOC2B localization to EVs and impacts its tumor-suppressive properties. Altogether, we propose that EV-mediated DOC2B transfer may be helpful for the management of CC.

## Materials and methods

### Cell culture, transfection, and generation of stable cell lines

We used a human cervix carcinoma cell line (SiHa and HeLa) obtained from ATCC, USA. We authenticated SiHa and HeLa cells by STR profiling before using them for experiments. SiHa cells inherently do not express DOC2B. We ectopically expressed DOC2B in SiHa cells via a retroviral-mediated transduction approach to generate SiHa cells overexpressing DOC2B gene (DOC2B cells), as published earlier^23^. The retrovirally transduced vector-transfected SiHa cells served as the control cells. The cells were routinely grown at 37 °C with 5% CO_2_ in Dulbecco’s modified Eagle’s medium (DMEM) supplemented with 10% heat inactivated fetal bovine serum (FBS). We preincubated the cells with 10 μM BAPTA-AM (Sigma-Aldrich, USA) for 1 h for calcium depletion studies^18^.

### Visualization of extracellular vesicle by live imaging

We transfected DOC2B and control cells grown in the confocal grade 35 mm Petri plate with mRFP Rab5a plasmid (Addgene, USA) using Lipofectamine 3000 (Invitrogen, USA). We performed live imaging using an SP8-DMi8 laser scanning confocal microscope (Leica Microsystems, Germany). We used excitation at 584 nm, emission at 607 nm, and a 100 × oil immersion objective, LAS software, and ImageJ tool for imaging and analyzing images. We analysed 100 cells selected from 10 random fields to count the EVs.

### Isolation, purification, and characterization of extracellular vesicles

We cultured DOC2B and control cells with and without BAPTA exposure in serum-free DMEM for 24 h. The conditioned medium collected from four 15 cm cell culture plates was centrifuged at 300 × g for 10 min, 3000 × g for 20 min, and 10,000 × g for 30 min to remove cell debris^26^. Subsequently, we ultracentrifuged the conditioned medium at 100,000 × g for 90 min using a swinging bucket rotor (SureSpin 630/17) in a Sorvall™ WX 90 + ultracentrifuge (Thermo Scientific, USA). After discarding the supernatant, the EV pellet was washed with 2 ml of syringe-filtered ice-cold phosphate-buffered saline (PBS), followed by ultracentrifugation at 100,000 × g for 90 min. The EV pellet was resuspended in PBS, filtered through 0.22 μm syringe filters (Millipore, USA), and stored at -80 °C until use. We used NanoSight NS300 (Malvern Instruments, Malvern, UK) to characterize the EVs. NTA 3.4 software calculated the size distribution, mean size values, count, and standard deviations. The size of the EV was determined using a Malvern Zetasizer ZS90 (Malvern Instruments, UK) and DLS technique^27^.

### Western blot

We used radioimmunoprecipitation assay (RIPA) lysis buffer supplemented with EDTA-free protease inhibitor cocktail (Calbiochem, USA) and BCA kit (Sigma‒Aldrich, USA) for lysate preparation and quantification of proteins. For immunoblotting, we separated the proteins (25–35 µg) by SDS‒PAGE (10–12%), followed by transfer onto a nitrocellulose membrane (GE Healthcare, USA). After blocking the membranes with 5% nonfat dry milk or BSA for one hour, the membranes were washed and the primary antibodies were incubated overnight at 4 °C: DOC2B (1:5000; Proteintech, USA), CD63 (1:3000; Abcam, Cambrdige, UK), VIM, CDH2, (1:3000; Cloud Clone, USA], ZEB1, CDH1, p-AKT1 (Ser473), total AKT1, p-ERK1/2 (Thr202/Tyr204), total ERK1/2, c-MYC, Active-β-Catenin (Active-CTNNB1), Total β-Catenin (CTNNB1), GAPDH (1:5000; Cell Signaling Technology, USA), p21 (CDKN2A), p27 (CDKN1B), CCNE (1:3000; Santa Cruz Biotechnology, USA] and SNAI1, β-ACTIN (1:5000; ABclonal, USA) (Table 1). The membranes were washed with TBST and incubated with HRP-conjugated anti-mouse or anti-rabbit secondary antibodies (1:5000; Cell Signaling Technology, USA) for one hour at room temperature. Western Blots were visualized using SuperSignal™ West Pico Chemiluminescent Substrate (Thermo Fisher Scientific, USA). We recorded the images using Image Quant LAS 4000 (GE Healthcare, USA) and iBright™ FL1500 Imaging System (Thermo Scientific, USA) (Table 1).

**Table 1:** List of Primary and Secondary antibodies.

Antibody Name	Isotype	Working Dilutions	Manufacturer	Catalogue Number
DOC2B	Rabbit	1:5000	Proteintech, USA	20,574–1-AP
CD63	Rabbit	1:3000	Abcam, Cambridge, UK	ab217345
pERK1/2	Rabbit	1:5000	Cell Signaling Technologies, USA	4370S
ERK1/2	Rabbit	1:5000	Cell Signaling Technologies, USA	4695S
pAKT1	Rabbit	1:5000	Cell Signaling Technologies, USA	4060S
AKT1	Rabbit	1:5000	Cell Signaling Technologies, USA	4691S
CDH1	Rabbit	1:5000	Cell Signaling Technologies, USA	3195 T
CDH2	Rabbit	1:3000	Cloud Clone Corp., USA	PAB481Hu01
cMyc	Rabbit	1:5000	Cell Signaling Technologies, USA	5605 T
SNAI1	Rabbit	1:5000	ABclonal, USA	A5544
CCNE	Mouse	1:3000	Santa Cruz Technologies, USA	sc-247
ZEB1	Rabbit	1:5000	Cell Signaling Technologies, USA	3396 T
P16 (CDKN2A)	Mouse	1:3000	Santa Cruz Technologies, USA	sc-1661
P27 (CDKN1B)	Mouse	1:3000	Santa Cruz Technologies, USA	sc-1641
VIM	Rabbit	1:3000	Cloud Clone Corp., USA	PAB040Hu01
Active CTNNB1	Rabbit	1:5000	Cell Signaling Technologies, USA	8814 S
CTNNB1	Rabbit	1:5000	Cell Signaling Technologies, USA	8480 T
GAPDH	Rabbit	1:5000	Cell Signaling Technologies, USA	2118
β-Actin	Rabbit	1:10,000	ABclonal, USA	AC026
anti-rabbit IgG-HRP	Rabbit	1:5000	Cell Signaling Technologies, USA	7074S
anti-mouse IgG-HRP	Mouse	1:5000	Cell Signaling Technologies, USA	7076S

### Labeling of extracellular vesicle and assessment of its uptake in SiHa cells

SiHa cells were grown on a chambered coverslip with 8 wells (Ibidi, Germany) and serum starved for 24 h. EV (50 µg/mL) isolated from exposed and unexposed calcium-chelator, BAPTA in DOC2B and control SiHa cells were labeled with 10 µM BODIPY™ TR Ceramide (Invitrogen, USA) for 20 min at 37 °C. We removed the excess BODIPY™ TR ceramide using EV spin columns (MW 3000; Thermo Scientific, USA)^28^. Serum-starved SiHa cells were incubated with labeled EV in phenol red-free DMEM, washed two times with PBS, and performed live imaging using a Leica SP8-DMi8 microscope (Ex: ∼589 nm and Em: 616 nm) with a 63 × oil immersion objective.

### MTT assay

SiHa and HeLa cells (1X10^4^ cells/well) were cultured in a 96-well plate for 48 h with purified EV (10 µg, 25 µg, 50 µg, 100 µg, and 150 µg/mL) in 1% serum-containing medium. In SiHa, various doses of cisplatin (2.5 µM, 5 µM, and 10 µM) were co-incubated with 100 µg/ml EV isolated from DOC2B cells for 48 h in DMEM with 1% FBS to test the chemosensitization ability of DOC2B. After that, 20 µl of MTT (5 mg/ml) added to the cells and incubated for 4 h. We solubilized the formazan crystals using DMSO. A multimode reader measured the optical density at 570–630 nm and was used to measure cell viability^29^.

### Scratch wound healing assay

SiHa and HeLa cells cultured to 80% confluency in a 6-well plate were serum starved for 48 h. We made a linear scratch in the middle of the monolayer cells with a sterile microtip. We exposed the cells to DMEM containing 1% serum and EV (100 μg/mL for SiHa and 120 μg/mL for HeLa). The migration of cells into the scratch area and subsequent wound closure were monitored using a camera (Jenoptik AG, Germany) attached to a CKX41 microscope (Olympus, Japan) at the indicated time points. The migration rate was calculated by subtracting the scratch area at 0 h from scratch at each specified time point using the ImageJ program as previously described^18^.

### Colony formation assay

We exposed SiHa and HeLa cells (100 cells) to 100 µg/mL and 120 μg/mL EV in a 35 mm cell culture dish for two weeks. The entire medium was discarded and replaced with new medium containing EV (100 µg/mL and 120 μg/mL) once every 3 days. At the end of 2 weeks, colonies were stained with 0.1% crystal violet solution prepared in methanol for 10 min. The plates were washed with PBS to remove excess stains and photographed. Colonies with more than 50 cells were counted^18^.

### Actin phalloidin staining

SiHa and HeLa cells were incubated with 100 µg/mL and 120 µg/mL EVs in a serum-free medium for 48 h. The treated cells were washed trice with PBS after fixation with 4% paraformaldehyde (PFA, Sigma‒Aldrich, USA) for 5 min. The cells costained with TRITC-conjugated actin-phalloidin (1:200; 30 min; Sigma‒Aldrich, USA) and Hoechst-33342 (1:1000; 5 min; Himedia, India) were washed briefly with PBS and mounted using Vectashield (Vector Laboratories, USA) onto a microscope slide^23^. We used SP8-DMi8 confocal microscopy (Actin-phalloidin: 540/570 nm and Hoechst-33342: 340/510 nm) for imaging. The images were examined by utilizing LAS software and ImageJ to quantify the length and number of filopodia and lamellipodia^30^.

### Cell cycle analysis

SiHa cells were serum starved for 24 h in a 6-well plate. After washing with PBS, cells were maintained in DMEM containing 2% FBS and 100 µg/mL EV for 24 h. After fixing in 70% ethanol, the cells were washed and exposed to RNaseA (0.5 µg/ml, Sigma‒Aldrich, USA) and propidium iodide (10 μg/mL, 30 min, ∼535/615 nm, Sigma‒Aldrich, USA) staining (19). The percentage distribution of cells at different stages of the cell cycle was evaluated using FACSCalibur and Cell Quest software (BD Biosciences, USA)^30^.

### Anoikis

The 6-well cell culture dishes received 2 mL of polyHEMA solution (10 mg/ml) prepared using 95% ethanol. After air drying, the wells were rinsed trice with PBS and air dried. We cultured SiHa cells in polyHEMA-coated wells along with EVs (100 μg/mL) for 48 h. Cells were collected and stained with acridine orange (AO; 100 μg/ml) and ethidium bromide (EtBr; 100 μg/ml) [Sigma‒Aldrich, USA] in PBS for 5 min. Unbound dyes were removed by washing briefly with PBS. The stained cells were photographed in the coverslip using a 63 × oil immersion objective (AO: 500/526 nm and EtBr: 482/616 nm) connected to a Leica SP8-DMi8 microscope and analyzed using LAS software^23^.

### Imaging intracellular calcium and ROS

SiHa and HeLa cells were grown on an 8-well chambered glass slide (Ibidi, Germany) and incubated with 100 µg/mL and 120 µg/mL EVs for 24 h. For intracellular calcium estimation, the EV-exposed cells were stained with a staining solution [Fluo-3 AM (1 µM), pluronic F-127 (0.01%, Molecular Probes, USA) and glucose (1 mg/ml) prepared in HBSS] for 45 min at 37 °C. For ROS estimation, the exosome enriched-EV exposed cells were stained with 2′,7′–dichlorofluorescein diacetate (DCFDA, 5 µM, Sigma, India) for 30 min at 37 °C. After washing with HBSS or PBS, the cells imaged using a Leica SP8-DMi8 microscope (Fluo-3 AM Ex: 506 nm and Em: 526 nm and DCFDA Ex: 485 and Em: 530 nm) with a 63 × oil immersion objective. Intracellular calcium and ROS were estimated using the average fluorescence intensity of 100 cells selected randomly using LAS software^25^.

### Lipid droplet measurement

We exposed SiHa cells grown on coverslips to 100 µg/mL EV in a serum-free medium for 24 h. The treated cells were washed and fixed with 4% PFA solution for 5 min. The Nile Red staining solution (5 μg/mL) (ThermoFisher Scientific, USA) incubated for 10 min and Hoechst-33342 (Himedia, India) for 5 min were washed briefly with PBS and mounted using Vectashield (Vector Laboratories, USA) onto a microscope slide ^25^. The images were acquired using an SP8-DMi8 confocal microscope (Nile Red: 552/636 nm; Hoechst-33342: 340/510 nm) and LAS software.

### Estimation of malondialdehyde (MDA) level

SiHa cells exposed to 100 µg/mL EV in serum-free medium for 24 h were suspended in lysis buffer (20 mM Tris–HCl pH 7.5, 1 mM EDTA, 150 mM NaCl, 1% Triton X-100), centrifuged, mixed with PBS, and then treated with TCA (20%). It was then centrifuged at 10,000 rpm for 15 min, the solution was mixed with 1% thiobarbituric acid and incubated in a boiling water bath for 30 min. The color developed was measured using a multimode reader at 532 nm. The MDA levels were normalized against the total protein^25^.

### Senescence-associated β-galactosidase (SA-β-gal) assay

SiHa and HeLa cells were incubated with 100 µg/mL and 120 µg/mL EVs in a serum-free medium for 24 h. After 4% PFA fixation, the cells exposed to a solution containing 150 mM NaCl, 5 mM K3Fe (CN)6, 5 mM K4Fe (CN)6, 2 mM MgCl2, 30 mM sodium phosphate buffer, and X-Gal (1 mg/ml) at pH 6.0 to develop color at 37 °C. We used a DP80 camera and BX51 microscope (Olympus, Japan) to capture the images. The cells stained blue-green were scored as senescence-positive cells^18^.

### Metabolite profiling of EVs

The global metabolite profile of EVs collected from vector-transfected and DOC2B over-expressing cells was analyzed using an Agilent 6520 TOF–MS coupled with a 1200 series HPLC system (Agilent Technologies, USA). We sonicated the EV pellets in pre-chilled 100% methanol for metabolite extraction. The extracted metabolites were subsequently lyophilized and reconstituted in a solution composed of water and acetonitrile (95:5, vol/vol) with 0.1% formic acid (HCOOH)^31^. Further, the separation of metabolites was carried out using an Eclipse Plus C18 column, with a scan range set at 50–1700 m/z and a scan rate of 1.4 spectra per second. The data analysis was performed using the HMDB database (www.hmdb.ca/)^32^ and MetaboAnalyst 6.0 (www.metaboanalyst.ca/)^33^.

### Statistical analysis

All values are expressed as the mean ± S.D. Statistical analysis was performed via unpaired t-test or one-way ANOVA using GraphPad Prism software. Statistical results with p-values lower than 0.05 were considered significant. We performed all experiments in duplicate and independently repeated three times.

## Results

### Characterization of the extracellular vesicles isolated from control and DOC2B cells

**Fig. 1:**
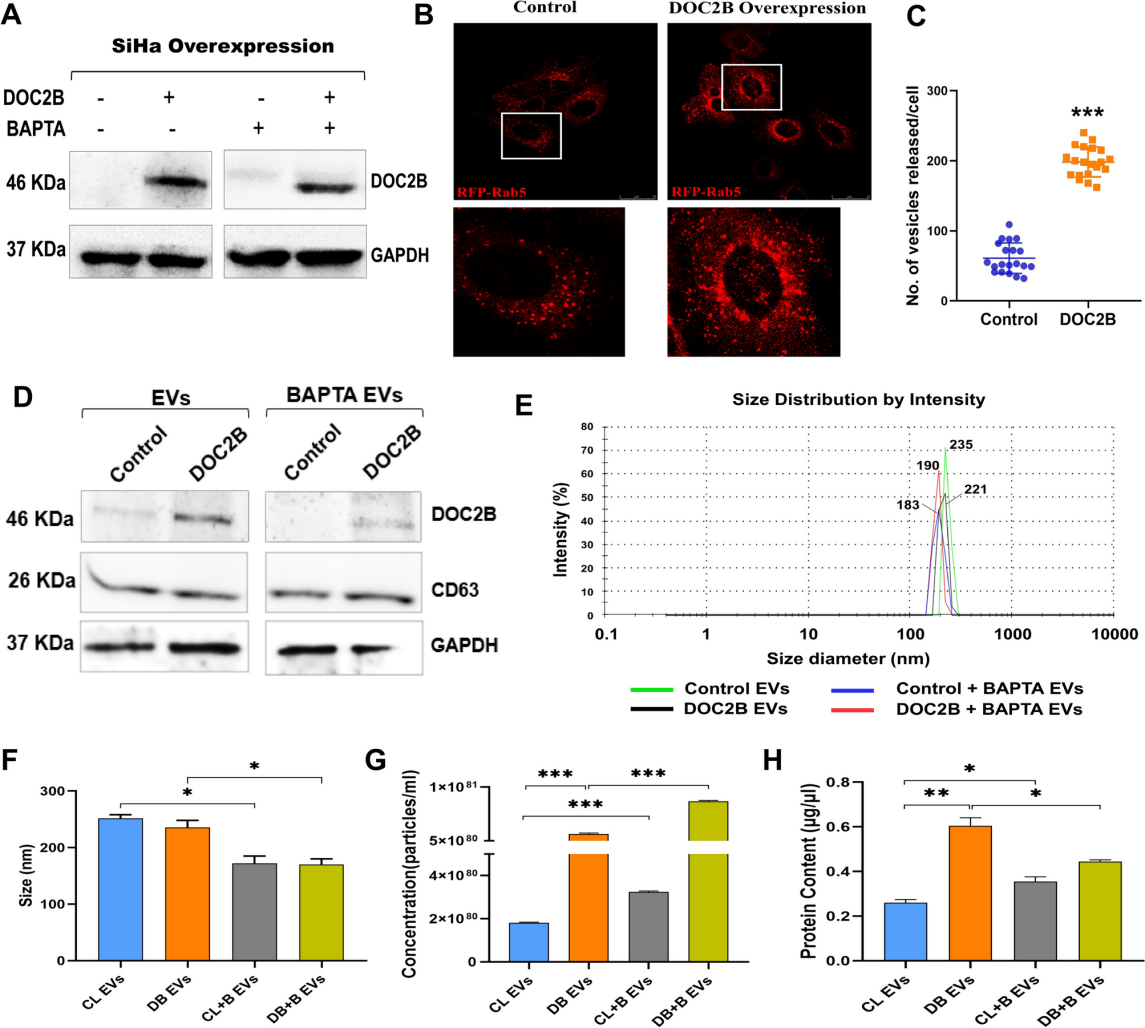
Characterization of EVs isolated from DOC2B overexpression cells** A**) Representative Western blot image confirming the ectopic expression of DOC2B in SiHa. **B**) Confocal microscopy images of control and DOC2B expressing SiHa cells transfected with RFP-tagged Rab5. **C**) Graph represents the number of vesicles released by control and DOC2B cells. **D**) DOC2B and extracellular vesicle-related marker (CD63) expressions were tested using Western blot analysis. **E)** Size distribution of EVs measured by Zetasizer. **F) & G)** The size and count analysis of EVs derived from control (CL EVs) and DOC2B (DB EVs) overexpressing SiHa cells before and after BAPTA treatment using NanoSight nanoparticle analyzer. **H)** EVs isolated from DOC2B showed higher protein concentration than EVs from vector transfected control SiHa cells. The asterisk indicates statistical significance (*P < 0.05, **P < 0.01, and ***P < 0.001). [DB EVs: DOC2B derived EVs; CL EVs: Control EVs; CL + B EVs: Control + BAPTA EVs; DB + B EVs: DOC2B + BAPTA EVs].

Due to promoter hypermethylation, DOC2B expression is downregulated in SiHa cells^23^. Hence, we ectopically expressed DOC2B in SiHa cells and confirmed its expression by western blotting (Fig. 1A). BAPTA treatment did not affect the total DOC2B level (Fig. 1A). The control and DOC2B-overexpressing cells transfected with Rab5A-RFP were subjected to live imaging using confocal microscopy (Fig. 1B). DOC2B-overexpressing SiHa cells showed significantly higher EV numbers than control cells (Fig. 1C).

### DOC2B localizes to extracellular vesicles

We evaluated the size and purity of EVs isolated from conditioned media of control and DOC2B SiHa cells with and without BAPTA treatment. SiHa cells do not express endogenous DOC2B. We performed Western blotting to test whether DOC2B localizes to EVs. We found that EVs isolated from DOC2B-overexpressing SiHa cells were positive for DOC2B (Fig. 1D). However, EVs from vector-transfected control SiHa cells did not show DOC2B expression. Interestingly, calcium chelation by BAPTA significantly reduced DOC2B expression in EVs isolated from DOC2B SiHa cells (Fig. 1D).

### Ectopic expression of DOC2B affects the properties of extracellular vesicles

We next investigated whether DOC2B manipulation impacts the properties of EVs. As per NTA, Zetasizer, and immunoblot analysis, the isolated EV size was 100–400 nM in diameter and was CD63 positive (Fig. 1D-1F). We showed that EVs isolated from DOC2B-SiHa cells were slightly smaller in diameter than those isolated from control cells. Intracellular calcium deprivation slightly reduced the EV size secreted from both control and DOC2B-overexpressing SiHa cells (Fig. 1E and 1F). The EV count and protein content were significantly higher in EVs isolated from DOC2B cells than in control cells (Fig. 1G and 1H).

We next investigated whether intracellular calcium deprivation affects the properties of EVs. Compared to untreated DOC2B SiHa cells, BAPTA treatment dramatically reduced EV size and protein content and increased its count. However, compared to untreated control-SiHa cells, BAPTA treatment drastically reduced the size of the EVs and increased their number and protein content (Fig. 1G and 1H).

### Extracellular vesicle-mediated transfer of DOC2B and its uptake by CC cells

**Fig. 2:**
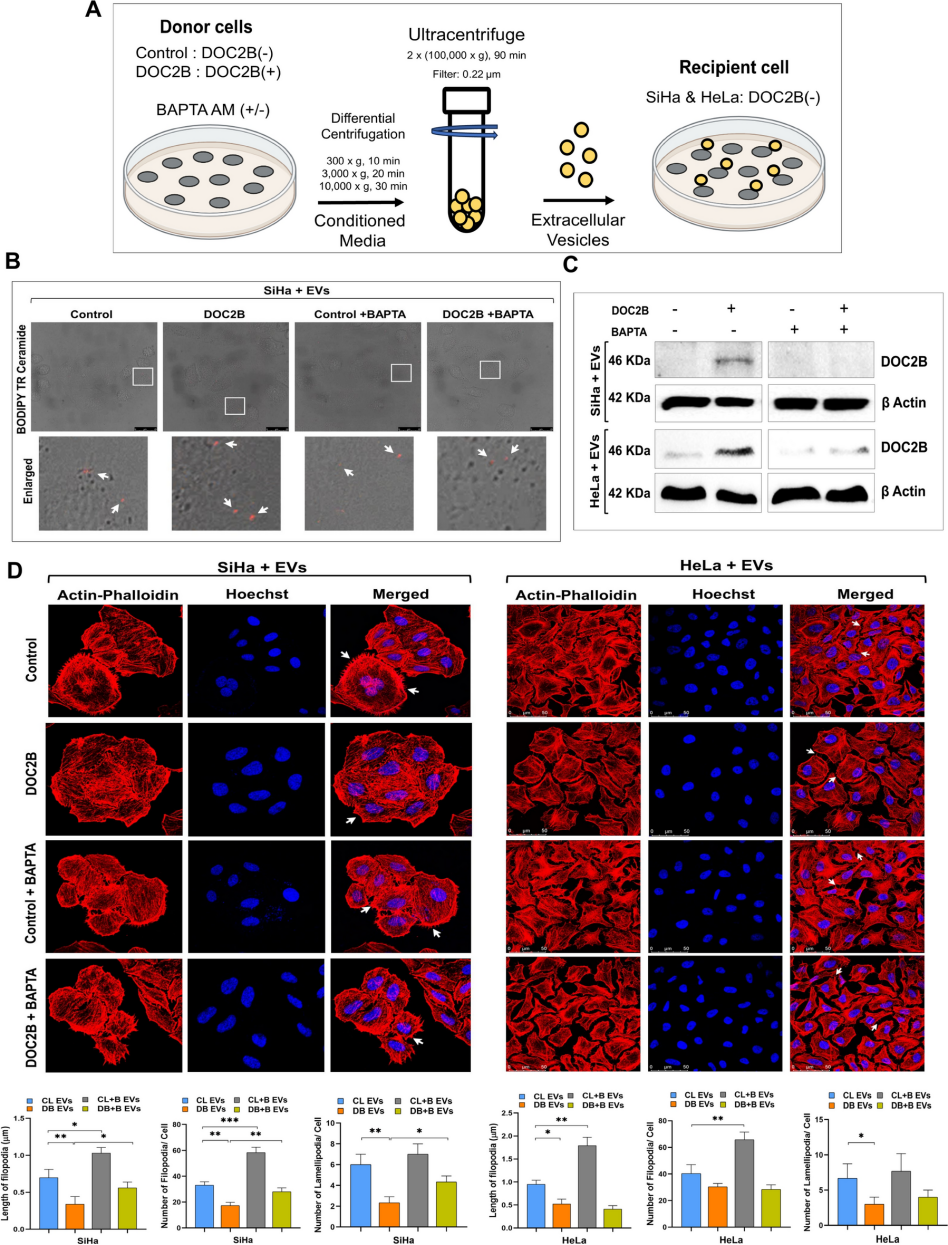
EV-mediated transfer of DOC2B to SiHa cells** A**) Schematic diagram showing EV harvesting by ultracentrifugation and delivery into recipient cells **B**) Western blot images showing the confirmation of DOC2B delivery via EV transfer. EVs isolated from calcium depletion failed to transfer DOC2B to recipient SiHa and HeLa cells. **C**) Represents confocal microscopy images showing the uptake of BODIPY™ TR Ceramide-labeled EVs collected from BAPTA exposed and unexposed DOC2B and control cells in SiHa. **D**) Represents actin phalloidin staining and confocal microscopy images of SiHa and HeLa cells that received EVs collected from vector transfected and DOC2B overexpressing SiHa cells with and without BAPTA treatment. EVs collected from DOC2B induced rearrangement of actin fibers, increased the length and number of filopodia in recipient cells than EVs collected from vector transfected control cells. These effects of DOC2B EVs on recipient cells were reversed upon transfer of EV from BAPTA exposed DOC2B cells. *P < 0.05 by independent Student’s t test was considered as statistically significant. [DB EVs: DOC2B derived EVs; CL EVs: Control EVs; CL + B EVs: Control + BAPTA EVs; DB + B EVs: DOC2B + BAPTA EVs].

We performed experiments with EVs isolated from control SiHa and DOC2B-overexpressing SiHa cells to confirm the observed biological effects in recipient cells via EV-mediated transfer of DOC2B (Fig. 2A). EVs derived from control and DOC2B SiHa cells were labelled with BODIPY™ TR ceramide to evaluate the efficiency of EV uptake by SiHa cells. SiHa cells were incubated with BODIPY™ TR Ceramide-labelled EVs (50 µg/mL), and EV uptake was examined by confocal microscopy. We observed several red fluorescent dots inside SiHa cells, suggesting the uptake of fluorescently labelled EVs (Fig. 2B).

To further confirm these findings, we incubated SiHa and HeLa cells with EVs (100 µg/mL and 120 µg/mL) isolated from control and DOC2B SiHa cells. The Western blot findings showed that recipient cells incubated with EVs isolated from DOC2B-SiHa cells showed DOC2B expression as opposed to recipient cells receiving EVs from control SiHa cells (Fig. 2C). Further, SiHa cells incubated with EVs isolated from BAPTA-exposed control-SiHa and DOC2B-SiHa cells did not show DOC2B expression, while HeLa cells slightly restore the DOC2B expression confirmed by Western blotting (Fig. 2C).

### Extracellular vesicle-mediated transfer of DOC2B induces morphological changes

We incubated SiHa and HeLa cells with EVs derived from control-SiHa and DOC2B-SiHa cells, followed by actin-phalloidin staining and confocal microscopy to assess the effects of DOC2B transfer on morphological changes in recipient cells. The recipient cells receiving EVs from control-SiHa cells showed significant morphological changes compared with cells receiving EVs from DOC2B-SiHa cells (Fig. 2D). SiHa cells receiving EVs from DOC2B-SiHa showed a significant reduction in the number and length of filopodia, while HeLa cells exhibited a substantial reduction in filopodia length and lamellipodia number compared to EVs from control-SiHa cells. Further, the transfer of EVs from BAPTA-treated control-SiHa and DOC2B-SiHa cells altered the actin cytoskeletal arrangements in recipient cells compared with unexposed cells. We observed a significant increase in the length and number of filopodia in SiHa, while no changes were observed in HeLa cells receiving EVs from BAPTA-treated control-SiHa and DOC2B-SiHa cells compared with the corresponding unexposed cells.

### Extracellular vesicle-mediated transfer of DOC2B inhibits key properties of CC cells

**Fig. 3:**
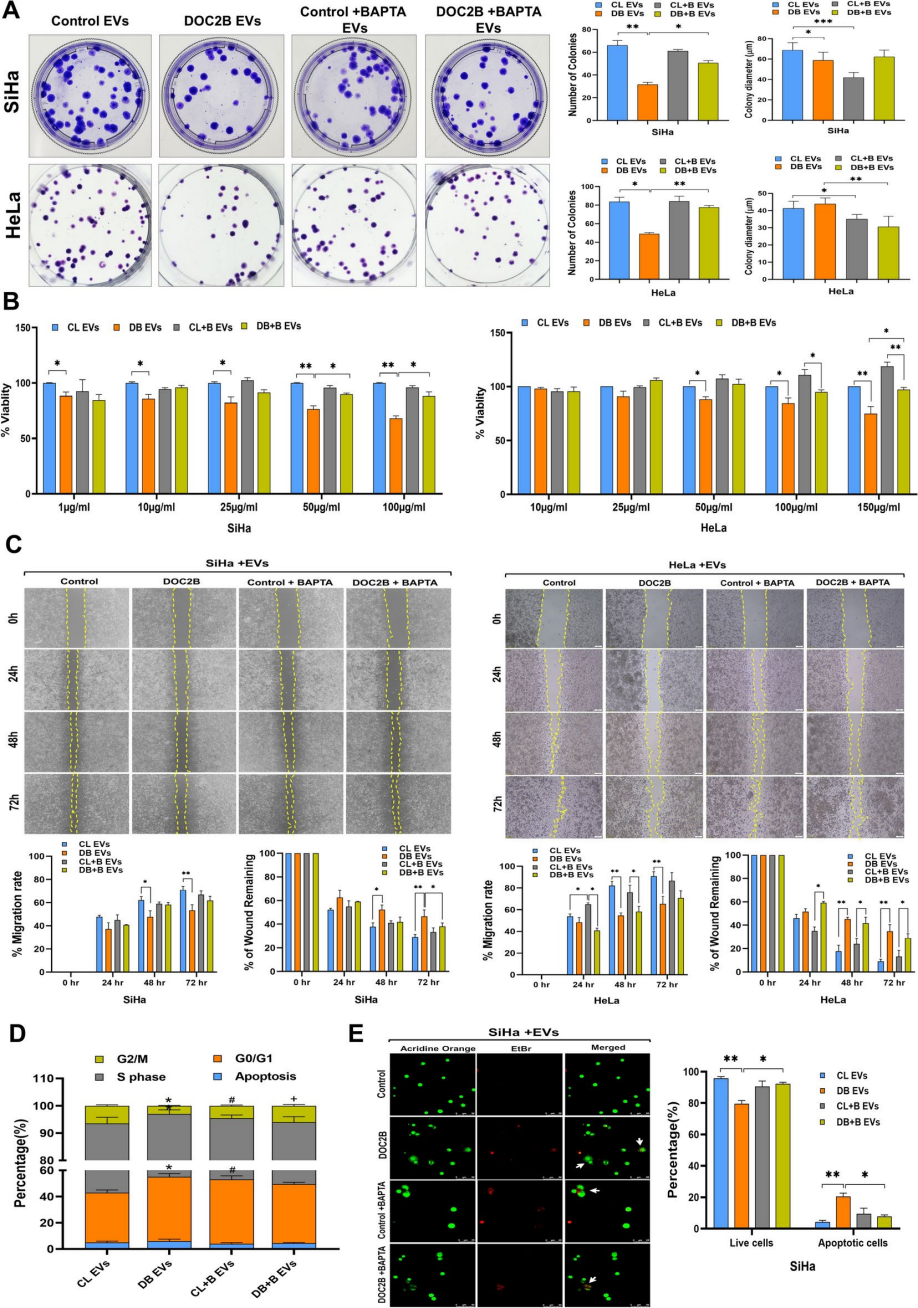
EVs from DOC2B elicited tumor suppressive properties in SiHa cells** A**) Represents the colony formation assay data from SiHa and HeLa cells receiving EVs harvested from vector transfected (Control) and DOC2B overexpressing SiHa cells with and without BAPTA exposure. The transfer of EVs from DOC2B overexpressing cells significantly inhibited the colony number in recipient cells compared to EVs harvested from vector transfected control cells. Calcium depletion reversed the tumor growth inhibitory properties of DOC2B EVs. **B**) The proliferation of SiHa and HeLa cells was significantly reduced in response to treatment with EVs collected from DOC2B-overexpressing cells. Calcium depletion reversed the anti-proliferative properties of DOC2B-EVs. **C**) Representative image of cell migration assay. DOC2B EVs significantly reduced the migration of recipient cells than control EVs. Calcium depletion reversed the migration inhibitory properties of DOC2B-EVs. **D)** DOC2B-EVs induced a strong G0/G1 to S-phase arrest in SiHa cells in comparison with control-EVs. BAPTA treated DOC2B-EVs relieved G0/G1 to S-phase arrest induced by untreated DOC2B-EVs. **E)** The anoikis-mediated cell death was significantly higher in SiHa cells exposed to DOC2B-EVs as opposed to control-EVs. Calcium depletion using BAPTA significantly reduced the Anoikis inducing ability of DOC2B-EVs. *P < 0.05 by independent Student’s t test was considered as statistically significant.

Next, we investigated the impact of EV-mediated DOC2B transfer on the biological properties of recipient cells by performing colony formation (growth), proliferation (MTT), and scratch (migration) assays. CC cells receiving EVs from DOC2B-SiHa cells showed a significant decrease in colony number compared with control-SiHa cells. The transfer of EVs from DOC2B-SiHa cells exposed to BAPTA treatment increased the colony number compared to CC cells that received EVs from DOC2B-SiHa cells unexposed to BAPTA (Fig. 3A).

We incubated SiHa and HeLa cells with multiple concentrations of EVs (1 µg/mL, 10 µg/mL, 25 µg/mL, 50 µg/mL, 100 µg/mL, and 150 µg/mL) for 48 h for cell proliferation analysis by MTT assay (Fig. 3B). DOC2B-EV exposure significantly inhibited the proliferation and migration of CC cells more than the control-EV exposure. However, BAPTA-treated DOC2B EVs significantly increased the proliferation and migration of CC cells more than unexposed DOC2B EVs (Fig. 3C). The present findings are consistent with our previous data that showed a negative effect of ectopic expression of DOC2B on the growth, proliferation, and migration of CC cells^18^.

### DOC2B extracellular vesicles induced cell cycle arrest and promoted anoikis

Cell cycle analysis using PI staining and flow cytometry showed robust G0/G1 to S phase arrest in the presence of DOC2B-EVs compared with control-EVs (Fig. 3D). Incubation of SiHa cells with DOC2B-EVs significantly increased the G0/G1 population (50% vs 38%) of cells and reduced S-phase cells (40% vs 51%) compared to control-EV exposed SiHa cells (Fig. 3D). Interestingly, incubation with EVs isolated from BAPTA-exposed DOC2B-SiHa cells reversed the cell cycle arrest exerted by EVs from BAPTA-negative DOC2B-SiHa cells. BAPTA treatment significantly decreased G0/G1 phase (42% vs 49%) and increased S-phase cells compared with (47% vs 42%) EVs isolated from BAPTA-negative DOC2B-SiHa cells (Fig. 3D). SiHa cells that received treatment with DOC2B-SiHa EVs produced more anoikis than control-SiHa EVs. EVs from DOC2B-SiHa cells were much less able to induce anoikis after exposure to BAPTA (Fig. 3E).

### DOC2B extracellular vesicle increases cytoplasmic ROS and calcium levels

**Fig. 4:**
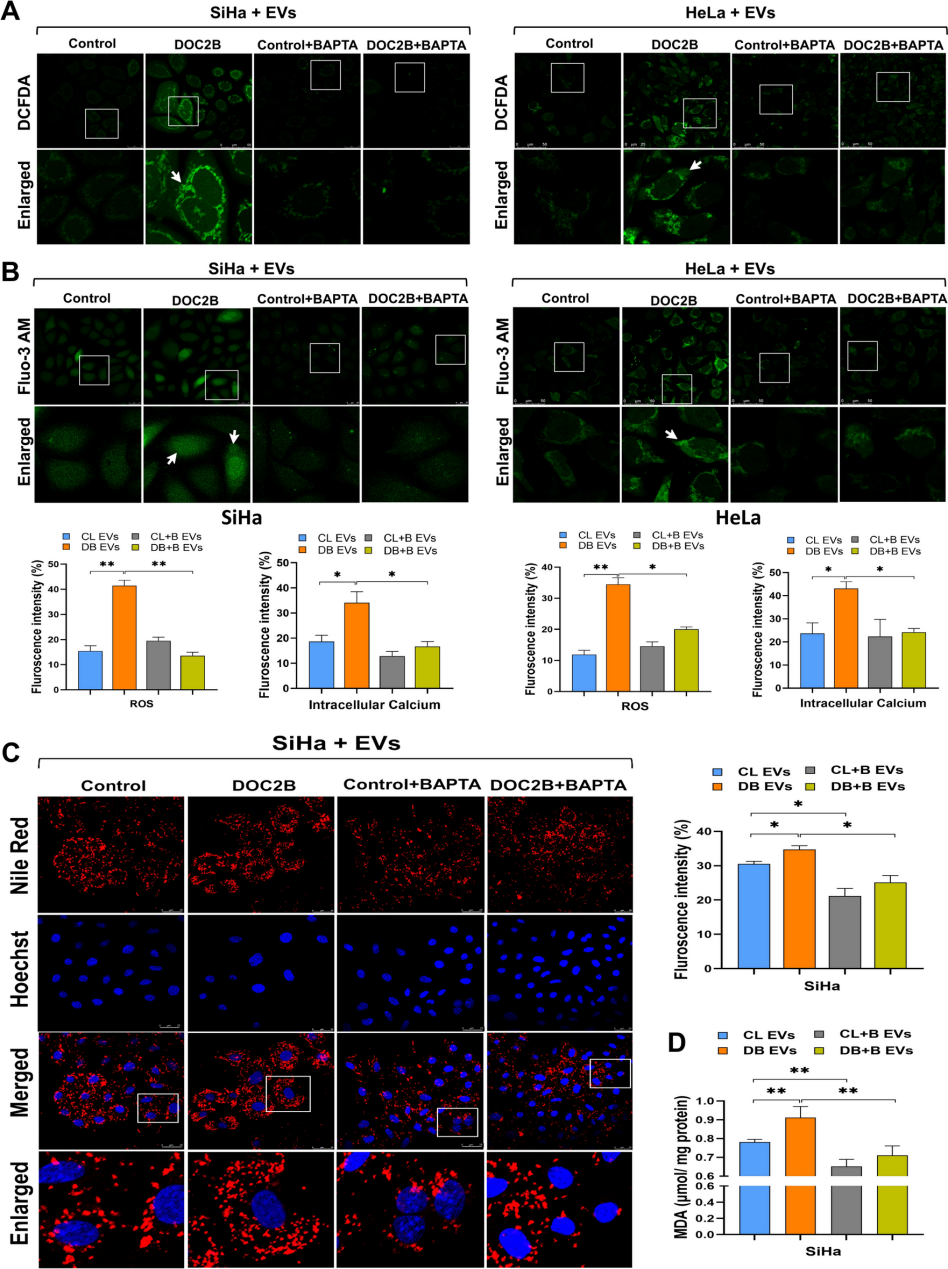
DOC2B EVs alters intracellular ROS, calcium, and lipid levels in SiHa** A**) Confocal microscopy images of SiHa and HeLa cells transferred with EVs from control and DOC2B overexpressing SiHa cells. DOC2B-EV transfer significantly elevated intracellular ROS level than EVs from control cells in SiHa and HeLa cells. DOC2B-EVs from calcium depleted DOC2B-SiHa reversed ROS inducing ability of unexposed DOC2B-EVs in recipient cells. **B**) In SiHa and HeLa cells, DOC2B-EV transfer significantly elevated intracellular calcium level than EVs from control cells. DOC2B-EVs from calcium depleted DOC2B-SiHa reversed intracellular calcium elevating ability of EVs collected from unexposed DOC2B cells. Bar graphs illustrate the ROS and calcium level in SiHa and HeLa cells exposed to DOC2B-EVs, control-EVs and EVs from calcium depleted control and DOC2B expressing SiHa cells. **C**) Representative images of Nile red stained SiHa cells exposed with DOC2B-EVs, control-EVs and calcium-depleted EVs. The lipid droplets accumulation was significantly higher in SiHa transferred with DOC2B-EVs as opposed to control EVs. Calcium depletion by BAPTA significantly lowered the lipid droplets in SiHa cells. **D)** SiHa cells receiving the DOC2B-EVs showed a higher LPO rate than Control-EVs. EVs from DOC2B overexpressing cells pre-treated with BAPTA showed significantly lower LPO rate than EVs from untreated DOC2B overexpressing cells. *P < 0.05 by independent Student’s t test was considered as statistically significant. [DB EVs: DOC2B derived EVs; CL EVs: Control EVs; CL + B EVs: Control + BAPTA EVs; DB + B EVs: DOC2B + BAPTA EVs].

We analyzed the effect of EVs on intracellular ROS and calcium via DCFDA and Fluo 3-AM staining and confocal microscopy. CC cells incubated with EVs from DOC2B-SiHa cells showed significantly higher basal levels of intracellular ROS and calcium than those incubated with EVs from control-SiHa cells (Fig. 4A and 4B). Incubation of recipient cells with BAPTA-treated DOC2B-SiHa EVs significantly decreased intracellular ROS and calcium (Fig. 4A and 4B). Intracellular ROS and calcium levels did not change considerably upon incubation with EVs isolated from control-SiHa, and BAPTA-exposed control-SiHa and DOC2B-SiHa cells.

### DOC2B extracellular vesicles promoted lipid droplet accumulation and lipid peroxidation

We estimated the lipid droplet accumulation and lipid peroxidation rate of SiHa cells upon exposure to EVs purified from DOC2B-SiHa and control-SiHa cells. Intracellular lipid droplet accumulation and the lipid peroxidation rate significantly increased in SiHa cells in response to DOC2B-SiHa EV exposure compared with control-SiHa EVs (Fig. 4C and 4D). Incubation with EVs from BAPTA-exposed DOC2B-SiHa and control-SiHa cells decreased the lipid droplet and lipid peroxidation rates of SiHa cells (Fig. 4C and 4D).

### Extracellular vesicles from DOC2B-SiHa cells reduced the expression of proliferation and EMT markers

**Fig. 5:**
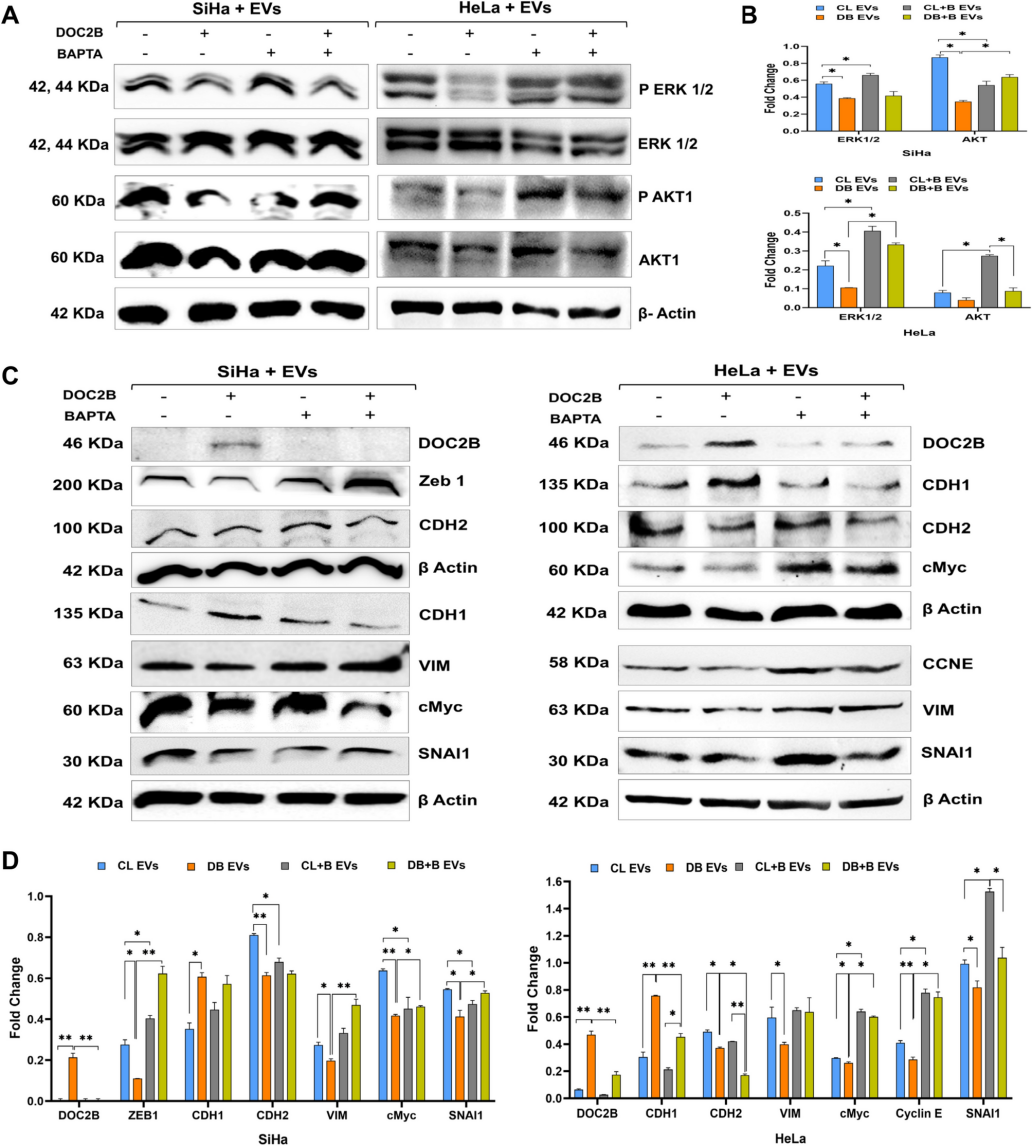
DOC2B-EVs inhibit epithelial to mesenchymal transition (EMT) pathway members **A**) Representative Western blot images show a reduction of AKT1 and ERK1/2 phosphorylation in SiHa and HeLa treated with DOC2B-EVs compared to control EVs. Calcium depletion relieved the inhibitory effect of DOC2B-EVs on AKT1 and ERK1/2. **B**) The bar graph represents the results of the densitometric analysis of the Western blot. **C**) Western blot showing the upregulation of CDH1 and downregulation of CDH2, VIM, ZEB1, SNAI1, CCNE and c-MYC in SiHa and HeLa cells exposed to DOC2B-EVs. **D**) Bar graph showing the quantitative analysis of expression of DOC2B, CDH1, CDH2, VIM, ZEB1, SNAI1, CCNE and c-MYC in recipient cells exposed to DOC2B-EVs as opposed to control-EVs. On the contrary, DOC2B-EVs from BAPTA-treated DOC2B cells enhanced the expression of ZEB1, VIM, c-MYC, CCNE and SNAI1 compared to untreated EVs from untreated cells. DOC2B-EVs collected from BAPTA treated DOC2B overexpressing cells and failed to transfer DOC2B to recipient cells. *P < 0.05 indicates statistical significance.

In SiHa cells, EV treatment from DOC2B-SiHa cells downregulated the active form of AKT1 and ERK1/2 without significantly changing total protein levels compared with EVs from control-SiHa cells. In HeLa cells, only active ERK1/2 was significantly downregulated, while AKT1 levels slightly decreased (Fig. 5A). Calcium-deprived EVs from DOC2B-SiHa cells upregulated the active form of AKT1 and ERK1/2 expression (Fig. 5B). We confirmed the restoration of DOC2B expression in SiHa and HeLa cells upon exposure with EVs from DOC2B-SiHa cells (Fig. 5C). Transfer of EVs from BAPTA-exposed DOC2B-SiHa cells to SiHa cells did not restore DOC2B expression, while a slight restoration was observed in HeLa cells (Fig. 5C). Remarkable upregulation of CDH1 and downregulation of CDH2, VIM, ZEB1, SNAI1, CCNE, and c-MYC were also noted in recipient cells upon treatment with EVs from DOC2B-SiHa cells compared with control-SiHa cells (Fig. 5D). Furthermore, the downregulation of VIM, ZEB1, and SNAI1 in SiHa cells in response to DOC2B-SiHa EVs was reversed upon exposure to BAPTA-treated DOC2B-SiHa EVs. Similarly, in HeLa cells, the downregulation of C-MYC, CCNE, and SNAI1 was reversed upon exposure to BAPTA-treated DOC2B-SiHa EVs (Fig. 5D).

### DOC2B extracellular vesicles induce cellular senescence

**Fig. 6:**
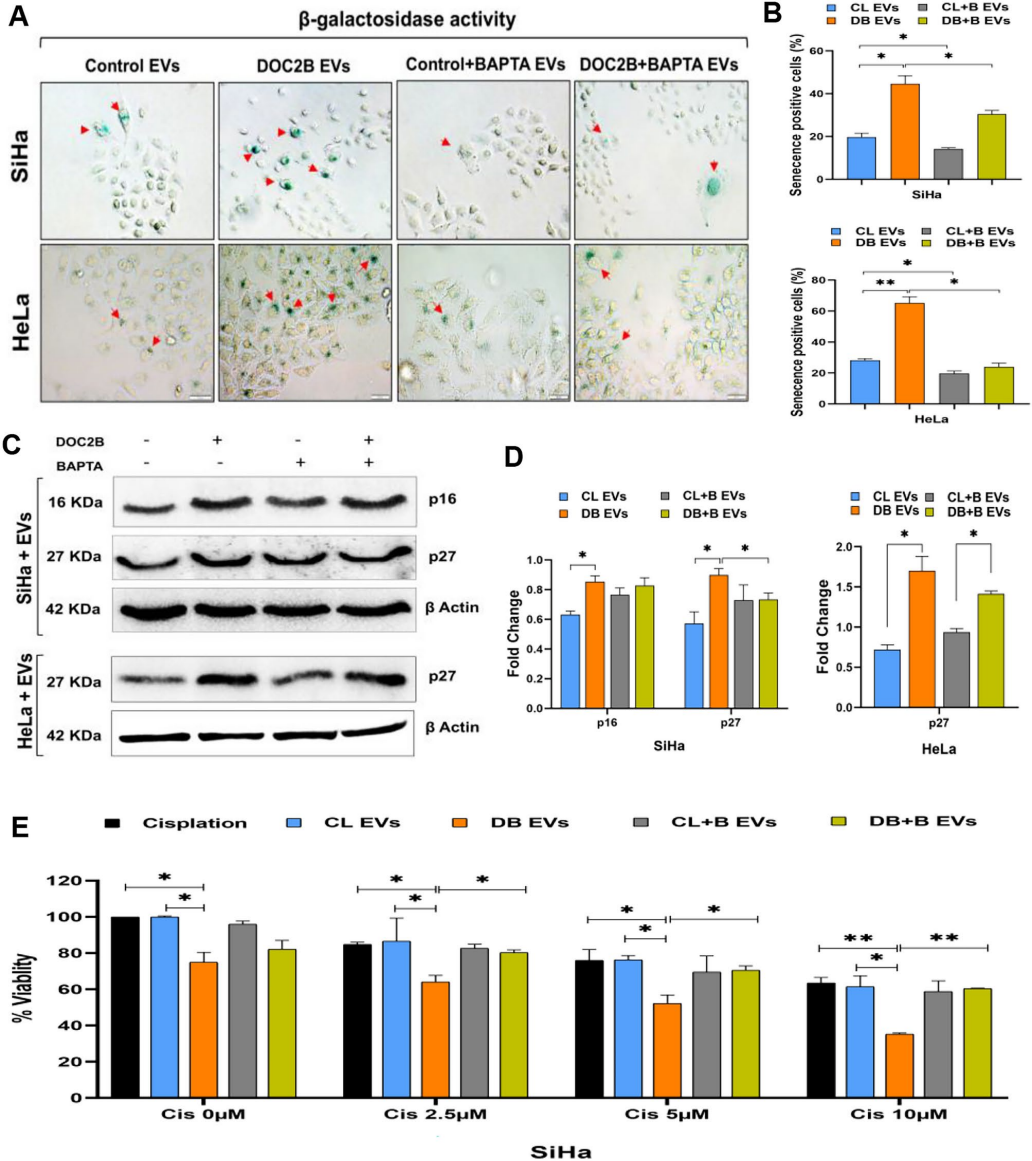
DOC2B-EVs induce senescence and enhance the cytotoxic effects of cisplatin in SiHa **A**) Representative image of SA-β gal staining (40 × magnification). SiHa and HeLa cells receiving DOC2B-EVs showed a higher senescence-positive cells than cells receiving EVs from control cells. Calcium depletion significantly reduced the senescence-inducing ability of DOC2B-EVs. **B**) The bar graph shows the quantitative analysis of SA-β-galactosidase-positive cells. **C**) The Western blot images showing p16 and p27 expression in SiHa and p27 expression in HeLa cells exposed to DOC2B-EVs and control-EVs. **D**) The bar graph shows an increased expression of p16 and p27 in recipient cells exposed to DOC2B-EVs than control-EVs. **E**) DOC2B-EVs transfer increased the cytotoxic effects of cisplatin in SiHa cells than control-EVs and BAPTA exposed DOC2B-EVs. *P < 0.05 indicates statistical significance.

SiHa and HeLa cells exposed to EVs isolated from DOC2B-SiHa showed significantly more senescence-positive cells than control-SiHa EVs (Fig. 6A and 6B). Additionally, exposure to DOC2B EVs increased the expression of p16 and p27 in SiHa cells and p27 in HeLa cells (Fig. 6C). EVs from BAPTA-treated DOC2B-SiHa and control-SiHa cells significantly reduced senescence in both SiHa and HeLa cells (Fig. 6D).

### Extracellular vesicle DOC2B increases the cytotoxic effects of cisplatin

We next tested the impact of EV DOC2B transfer on enhancing the cytotoxic effect of cisplatin on SiHa cells by MTT assay. For the cytotoxicity assay, we treated SiHa cells with only EVs, EVs with different concentrations of cisplatin, and BAPTA-treated EVs with varying concentrations of cisplatin. SiHa cell proliferation was significantly reduced upon cisplatin treatment compared with untreated cells (Fig. 6E). Treatment with EVs harvested from DOC2B-SiHa cells significantly inhibited SiHa cell proliferation more than treatment with EVs harvested from control-SiHa cells. We exposed SiHa cells to a combination of 100 µg/mL EVs with 2.5 µM, 5 µM, and 10 µM cisplatin. SiHa cells exposed to EVs from DOC2B-SiHa cells showed significantly higher cisplatin cytotoxicity than those exposed to EVs from control-SiHa cells in a dose-dependent manner (Fig. 6E). However, incubation of SiHa cells with EVs from BAPTA-positive DOC2B-SiHa cells significantly reversed the cytotoxic effects of cisplatin compared with incubation with EVs from BAPTA-negative DOC2B-SiHa cells (Fig. 6E).

### Localization of β-Catenin into EVs

**Fig. 7:**
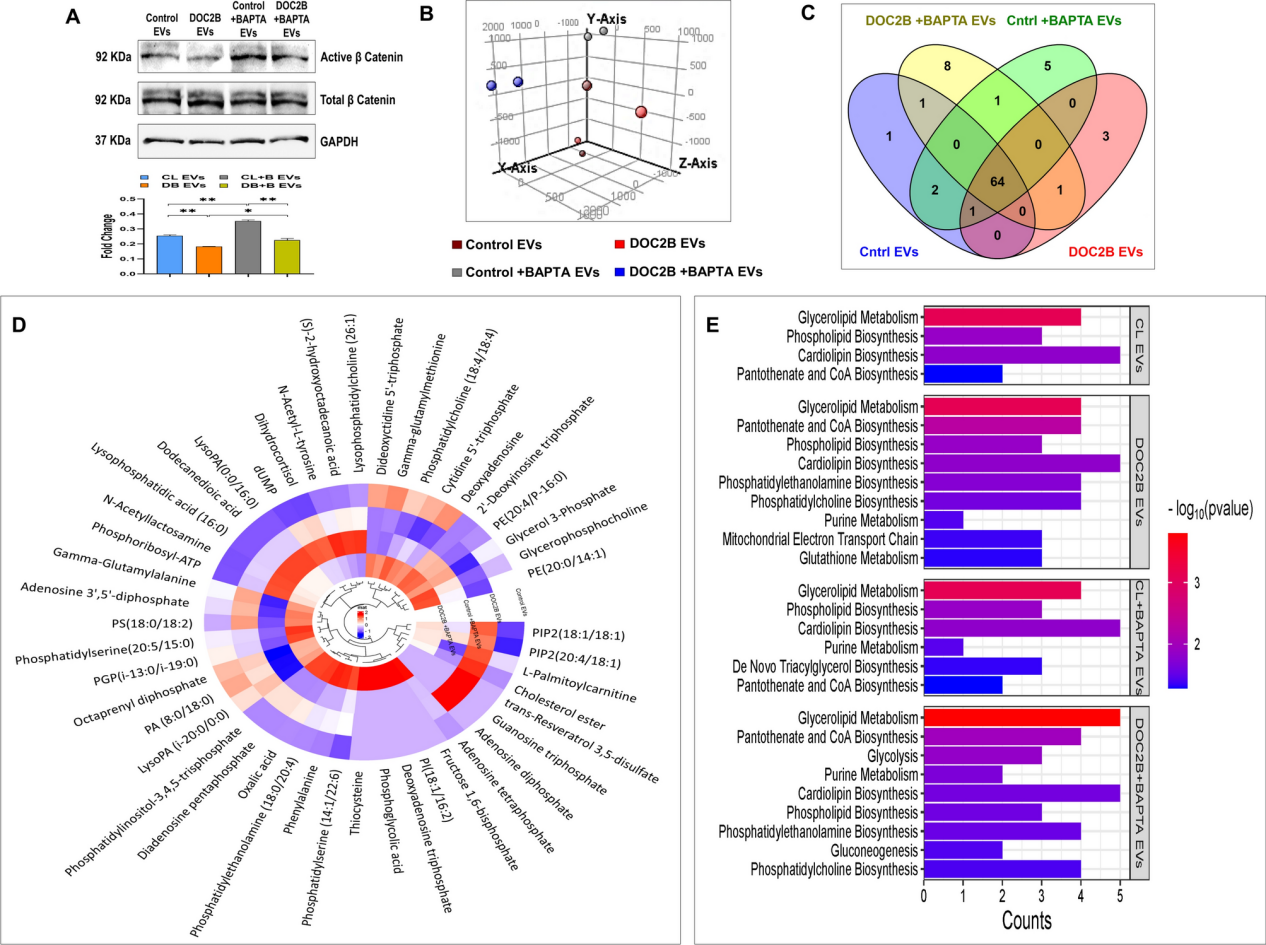
EVs derived from DOC2B alter the molecular cargo and global metabolite profile **A**) Western blot images showing active β-catenin expression in EVs collected from Vector-transfected and DOC2B-transfected SiHa cells and EVs from Vector-transfected and DOC2B-transfected cells exposed to BAPTA. **B**) Principal component analysis (PCA) was performed using EV metabolites from vector-transfected, DOC2B-transfected, and calcium-depleted vector-transfected and DOC2B-transfected SiHa cells. **C**) The Venn diagram represents the metabolite distribution between EVs. **D**) Circular cluster heatmap represents the differential abundance of EV metabolites from Vector-transfected, DOC2B-transfected, and calcium-depleted SiHa cells. **E**) The pathway enrichment analysis of EV metabolites from Vector-transfected, DOC2B-transfected SiHa, and calcium-chelated cells using MetaboAnalyst 6.0.

To further investigate how calcium influences EV cargo, we analyzed the expression levels of active β-Catenin in EVs collected from DOC2B overexpressing cells before and after calcium chelation using BAPTA (Fig. 7A). The results showed that active β-Catenin expression was significantly reduced in EVs collected from DOC2B-SiHa cells compared to control-SiHa EVs. Interestingly, EVs collected from BAPTA-treated DOC2B-SiHa showed an upregulation of active β-Catenin levels (Fig. 7A). Our previous work demonstrated that DOC2B inhibits the nuclear localization of active β-Catenin, compared to vector-transfected SiHa cells^24^. These findings indicate that calcium depletion can alter the cargo composition of EVs.

### DOC2B alters metabolite distribution in EVs

We performed a global metabolomic profiling of EVs secreted by control-SiHa and DOC2B-SiHa cells in the presence and absence of BAPTA. DOC2B re-expression significantly altered the metabolite composition in the EVs. PCA analysis of metabolite profiles revealed a clear separation between EVs from control-SiHa and DOC2B-SiHa cells, as well as between BAPTA-treated control-SiHa and DOC2B-SiHa cells (Fig. 7B). The total number of metabolites identified was 69 and 71 in EVs from control-SiHa and DOC2B-SiHa cells, respectively, and 73 and 76 in EVs from BAPTA treated control-SiHa and DOC2B-SiHa cells (Fig. 7C).

Metabolites such as Phosphatidylinositol 4,5-bisphosphate (PIP2), L-Palmitoylcarnitine, Cholesterol ester, Guanine triphosphate, and Adenosine diphosphate were highly abundant in DOC2B-SiHa EVs compared to control-SiHa EVs, and their levels were significantly reduced in EVs collected from BAPTA treated DOC2B-SiHa. Conversely, EVs collected from BAPTA-treated DOC2B-SiHa displayed higher levels of metabolites, notably Phosphatidylinositol (3,4,5)-trisphosphate (PIP3), Diadenosine pentaphosphate, Oxalic acid, Phosphatidylethanolamine, Phenylalanine, Phosphatidylserine, Phosphatidylglycerol phosphate (PGP), Phosphatidylethanolamine (PE), and Glycerophosphocholine compared to untreated DOC2B-SiHa EVs. Additionally, BAPTA-treated DOC2B-SiHa EVs contained unique metabolites such as Thiocysteine, Phosphoglycolic acid, Phosphatidylinositol (PI), Deoxyadenosine triphosphate, Fructose bisphosphate, and Adenosine tetraphosphate (Fig. 7D). These data collectively suggested that BAPTA exposure can modify the EV cargos.

### Metabolite set enrichment analysis

**Fig. 8:**
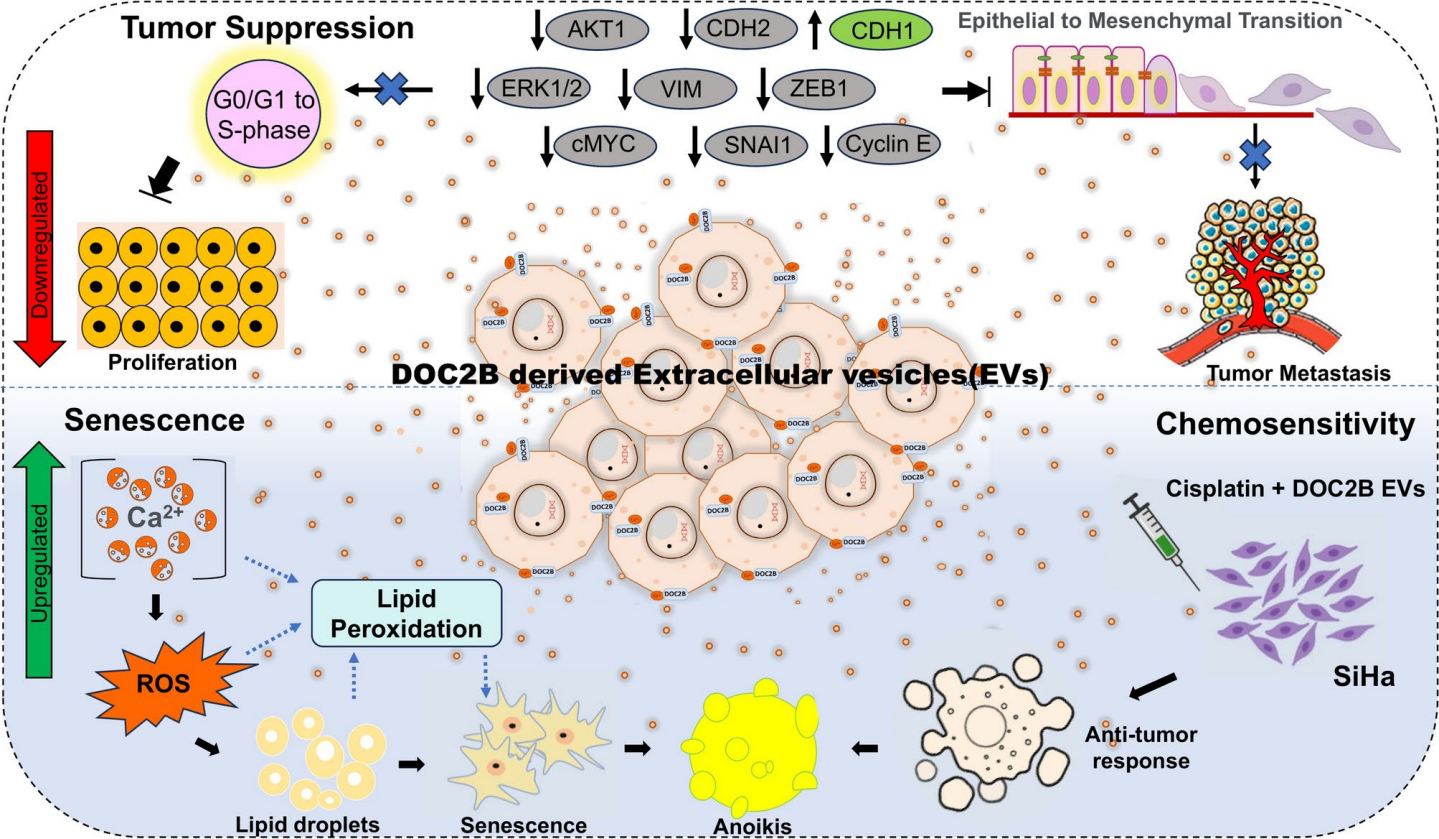
Proposed model for Extracellular vesicle DOC2B mediated tumor suppressive function Extracellular vesicle DOC2B regulates EMT and senescence process via inhibiting proliferation, migration, growth and promotes cell cycle arrest (G0/G1 to S-phase), intracellular ROS, lipid peroxidation and chemosensitivity in CC.

The top enriched KEGG pathways (p < 0.05) between control-SiHa and DOC2B-SiHa cells under untreated and BAPTA-treated conditions are illustrated in Fig. 7E. KEGG pathways provide a comprehensive, visual, and functional representation of molecular networks, aiding in the understanding of complex biological systems. Pathways such as pantothenate and CoA synthesis, phosphatidylethanolamine and phosphatidylcholine biosynthesis, mitochondrial electron transport chain, purine metabolism, and glutathione metabolism were highly enriched in DOC2B-SiHa EVs compared to control-SiHa EVs. In contrast, BAPTA-treated DOC2B-SiHa EVs showed an increased association with pathways such as glycerolipid metabolism, glycolysis, purine metabolism, and gluconeogenesis compared to untreated DOC2B-SiHa EVs. Notably, metabolites in DOC2B-SiHa EVs were enriched with lipids and fatty acid derivatives, which are critical for lipid biosynthesis. Following calcium chelation, DOC2B-SiHa EVs exhibited an enhanced release of metabolites linked to pathways connected with energy production, such as glycolysis and gluconeogenesis (Fig. 7E). Collectively, our data demonstrates that DOC2B re-expression significantly alters the metabolome of EVs. Further, calcium chelation alters the metabolite composition of EVs. Figure 8 provides an overview of the key findings of our study.

## Discussion

CC is the 4^th^ most frequent gynecological cancer globally and is a key public health concern in many developing and underdeveloped countries^1,2^. Vaccination against HPV and effective implementation of PAP screening reduced the CC burden in many well-developed countries ^4^. In developing and underdeveloped countries, inadequate access to these preventive measures may have contributed to the late diagnosis of CC. Additional factors, including inadequate access to treatment facilities and lack of effective treatment for advanced cancer, may contribute to higher mortality in these countries. Soluble factors released from EVs into the tumor microenvironment may play an active role in tumorigenesis^34^. Cargo manipulation studies have indicated that EVs may have tumor-promoting or tumor-inhibiting characteristics depending on their cargo^35,36^. Loading EVs with tumor-suppressive microRNAs, proteins, long noncoding RNAs, or drugs is being investigated for cancer management^37^. Thus, it is crucial to understand the cargo content and identify mechanisms for the clinical management of CC. In this study, we investigated how DOC2B localized EVs when transferred to DOC2B-negative SiHa cells, exert tumor suppressive properties using an in vitro model system.

Our previous studies demonstrated DOC2B as a metastatic suppressor downregulated in CC patients and cell lines by gaining promoter methylation^23^. DOC2B reintroduction in CC cells inhibited EMT and WNT signaling and induced senescence^18,24^. The tumor suppressive function of DOC2B correlated with structural and functional alterations in mitochondria^25^. DOC2B contains calcium sensing and binding domains and may depend on intracellular calcium for several functions, including tumor-suppressive properties. Calcium plays a crucial role in the biogenesis of EVs, particularly in pathways involving the ESCRT (Endosomal Sorting Complex Required for Transport) machinery and exocytosis. Calcium influx has been shown to enhance the budding of EVs from multivesicular bodies and the plasma membrane^38^. Therefore, we investigated whether EVs isolated from DOC2B-SiHa cells exhibit tumor suppressive properties compared to EVs from control-SiHa cells by EV transfer experiments.

We first showed that EVs harvested from DOC2B-SiHa and control-SiHa cells are taken up by SiHa and HeLa cells by Western blot and confocal microscopy. EVs from DOC2B-SiHa cells significantly inhibited the growth, proliferation, and migration of SiHa and HeLa cells compared to EVs from control-SiHa cells. DOC2B-SiHa EVs showed a strong G0/G1 to S-phase arrest, reversed anoikis resistance, and induced senescence compared to control-SiHa EVs in SiHa cells. We noted a reduction in the number and length of filopodia, along with morphological changes in SiHa cells, while HeLa cells showed a decrease in filopodia length and lamellipodia number upon receiving EVs from DOC2B-SiHa cells than control-SiHa cells. These observations were consistent with our previous studies that showed that DOC2B inhibits the growth, proliferation, and migration of SiHa cells via induction of cell cycle arrest, anoikis, actin cytoskeletal rearrangement, and reduced filopodia in CC^18^. Our study shows that DOC2B can be transferred to SiHa and HeLa cells via EVs without compromising its tumor-suppressive functions. Therefore, the tumor-suppressing properties of DOC2B-SiHa EVs in SiHa and HeLa cells may be partly contributed by EV DOC2B. More detailed preclinical studies using EVs containing DOC2B are required to translate the findings into clinical use.

It has been demonstrated that changes in second messengers—ROS and intracellular calcium flux—play a role in cancer development^39^. ROS and intracellular calcium flux increases are associated with tumor-suppressive properties in certain cancers^40–42^. Elevation in intracellular ROS can induce lipid peroxidation to promote apoptosis^42^. Besides, oxidative stress via enhanced intracellular ROS can induce senescence in several cell types^43^. Previously, we showed that ectopic expression of DOC2B induced oxidative stress and elevation in intracellular calcium, lipid droplet accumulation, and lipid peroxidation to promote anoikis and senescence^18,25^. The higher anoikis and senescence-inducing ability of DOC2B-SiHa EVs could be due to the oxidative stress and elevation in intracellular calcium caused partly by DOC2B present in the EVs. Based on the findings from our study, we propose that intracellular calcium overload may be responsible for DOC2B-induced lipid droplet accumulation, oxidative stress, and lipid peroxidation.

CC shows frequent activation of the PI3-AKT, MAPK, and EMT pathways^7^. These pathways, when activated, promote growth, proliferation, migration, invasion, and therapy resistance in CC. Thus, targeting the PI3-AKT, MAPK, and EMT pathways could help CC management. In contrast, senescence induction is considered a tumor-suppressive mechanism in CC^44^. Our study shows that EVs from DOC2B suppress active AKT1 and ERK1/2 and members of the EMT signaling pathway, including CDH2, VIM, ZEB1, SNAI1, CCNE and c-MYC, with simultaneous upregulation of CDH1. These findings align with our previous results that showed an inhibitory effect of DOC2B on the active form of AKT1, ERK1/2, and members of the EMT pathway^18^. The reduction in active AKT1, c-MYC, and ERK1/2 may have contributed to the reduced proliferation and growth of SiHa and HeLa cells upon receiving DOC2B-SiHa EVs. The upregulation of CDH1 and downregulation of CDH2, VIM, ZEB1, SNAI1, CCNE, and c-MYC may inhibit migration, cell cycle and EMT. Therefore, we propose that reintroducing DOC2B via EVs can be employed as an approach to reduce active AKT1, ERK1/2, and EMT signaling.

We demonstrated that DOC2B can impact the localization of active β-Catenin into EVs. More importantly, intracellular calcium may be necessary for DOC2B-mediated control of β-Catenin localization in EVs. Further, we showed that the re-expression of DOC2B can change the composition of metabolites within EVs, especially the abundance of lipids, fatty acids, and steroids. EVs from DOC2B-SiHa are abundant with lipid derivatives such as PIP2, L-Palmitoylcarnitine, and Cholesterol ester compared to EVs from control-SiHa cells. Further, BAPTA-treated DOC2B-SiHa EVs secreted higher levels of phospholipid derivatives, organic acid derivatives, nucleoside, and nucleotide analogs than untreated DOC2B-SiHa EVs. Collectively, our data demonstrates that DOC2B re-expression and calcium depletion can significantly alter the metabolite composition of EVs.

Cisplatin is the most commonly used and successful chemotherapeutic agent against CC^7^. Cisplatin resistance is rising and leads to treatment failure^45,46^. Proteins, nucleic acids, and lipids in EVs can regulate signaling pathways to promote drug resistance in recipient cells. Hence, we assessed whether restoration of DOC2B can increase the cytotoxicity of cisplatin. The coincubation of EVs carrying DOC2B with varying concentrations of cisplatin increased the cytotoxicity of cisplatin more than the DOC2B deficient control EVs. Thus, combining cisplatin with EVs containing DOC2B could help enhance the cytotoxic effects of cisplatin.

Intracellular calcium regulates many signaling pathways that are critical for cellular functioning. Several studies have documented the association between deregulated calcium signaling and the acquisition of cancer hallmarks^47^. Hence, targeting calcium signaling is now an important emerging area of cancer research. Previously, we showed that intracellular calcium is critical for the tumor-suppressive properties of DOC2B in CC^18^. Hence, we investigated whether calcium depletion impacts the tumor-suppressive properties of EVs from DOC2B-SiHa cells. The reintroduction of DOC2B via EVs showed a significant elevation in intracellular calcium levels that correlated with the tumor-suppressive properties of DOC2B. Reversal of tumor suppressive function upon BAPTA treatment suggests that the rise in intracellular calcium seems to play a critical role in controlling the growth, proliferation, and migration of CC cells. Furthermore, calcium depletion may also have impacted on the composition of EV cargo and subsequent biological effects in recipient cells.

Our in-vitro findings demonstrate a strong tumor-suppressive effect of DOC2B-loaded EVs. Thus, DOC2B delivered via EV has an excellent possibility for clinical management of CC. Despite this, several challenges need to be addressed, such as problems connected with purification, determining the most efficient size, poor characterization, loading efficiency, and tumor targeting specificity. One of the critical considerations is the biodistribution of EVs. Effective delivery to tumor sites while avoiding off-target effects is a primary challenge in EV-based therapies. Targeting mechanisms, such as surface modification or ligand attachment to improve tumor-specific uptake, could enhance the precision of DOC2B-loaded EVs in cervical tumors. Another factor to consider is the clearance of EVs from the bloodstream. EVs are typically cleared by the liver, spleen, and kidneys, which could limit their therapeutic efficacy in reaching tumor sites. Strategies to extend EV circulation time and bioavailability can be achieved using engineering EVs with stealth properties or optimizing their lipid composition.

Additionally, the potential immunogenicity of DOC2B-loaded EVs requires thorough investigation. Although EVs are generally considered less immunogenic than synthetic nanoparticles, introducing foreign proteins or altered cargo content may elicit immune responses. Understanding repeated EV administration’s immune profile and safety is crucial to prevent adverse reactions. Preclinical animal models are needed to evaluate the in vivo efficacy, biodistribution, and safety of DOC2B-loaded EVs and translate our findings into clinical use. Moreover, combination strategies, such as co-administering DOC2B-loaded EVs with chemotherapeutic agents like cisplatin, may enhance treatment outcomes and offer new avenues for personalized cervical cancer therapies.

## Conclusion

We reported for the first time that DOC2B is localized to EVs, and its transfer via EVs suppressed the aggressive behavior of SiHa and HeLa cells. EV transfer of DOC2B, and associated tumor suppressive functions could be linked to (i) morphological changes, senescence, anoikis, G0/G1 to S phase arrest, ROS, intracellular calcium, lipid droplet accumulation, and lipid peroxidation, and (ii) EMT inhibition and ERK1/2 activity reduction in recipient CC cells. EV DOC2B alters global metabolite profile and enhanced the cytotoxic effects of cisplatin. Intracellular calcium may be critical for the tumor suppressive properties of EV-DOC2B. Our study highlights that intracellular calcium may be vital for determining the cargo content of EVs. Evidence from the present study shows that functional DOC2B delivery by EVs could be helpful in CC treatment. A combination of DOC2B and cisplatin could be a valuable strategy for the clinical management of CC.

## Ethical approval

Ethical approval was not required for the present study.

## Data Availability

The authors declare that all other data supporting the findings of this study are available within the article and its supplementary information files.
